# From freshman to graduate: longitudinal analysis of student health status in sport and health sciences

**DOI:** 10.1186/s12889-026-29582-5

**Published:** 2026-09-21

**Authors:** Klaus Christian Haggenmüller, Barbara Reiner, Nils Olson, Jochen Weil, Thorsten Schulz, Renate Maria Oberhoffer, Sebastian Freilinger

**Affiliations:** 1 Chair of Preventive Pediatrics, Department Health and Sport Science, School of Medicine and Health, Technical University of Munich, Am Olympiacampus 11, Munich, 80809 Germany; 2 TUM University Hospital German Heart Center, Lazarettstrasse 36, Munich, 80636 Germany

**Keywords:** University students, Longitudinal study, Cardiorespiratory fitness, VO₂peak, Physical activity, Cardiovascular risk, Health promotion, Young adults

## Abstract

**Background:**

University years are considered a critical period for the development of long-term health behaviors. However, evidence on longitudinal health trajectories in students is limited, particularly regarding objectively measured physiological outcomes. This study aimed to examine changes in cardiorespiratory fitness, anthropometrics, blood pressure, and cardiovascular risk factors during undergraduate studies, as well as the influence of physical activity, sex, and age.

**Methods:**

This longitudinal observational study included 128 students (mean age at baseline 21.0 ± 2.1 years; 75% female) enrolled in sport science or health science programs at the Technical University of Munich. Participants underwent comprehensive physiological assessments at baseline and follow-up (mean interval 2.53 ± 0.60 years), including cardiopulmonary exercise testing, body composition analysis, blood pressure, and heart rate measurements, and a structured medical history interview supplemented with questions on smoking status, alcohol consumption, weekly training volume, and dietary patterns (omnivorous, vegetarian, or vegan). Paired-samples t-tests were used to compare baseline and follow-up measurements; additionally, linear mixed-effects models were used to evaluate longitudinal changes and predictors of individual trajectories.

**Results:**

In the unadjusted paired analysis, VO₂peak increased significantly from baseline to follow-up (*p* < .001, Cohen’s d = 0.434), accompanied by a reduction in resting heart rate (*p* < .001, Cohen’s d = 0.378), whereas maximal power output remained unchanged. Body weight, BMI, and body fat percentage remained largely stable, whereas muscle mass increased significantly in the unadjusted paired analysis (*p* = .006). However, after adjustment for sex, age, study program, and weekly training volume in the mixed-effects model, no statistically significant independent main effect of time on VO₂peak was observed. Higher weekly training volume was independently associated with higher VO₂peak, whereas sex, age, and study program did not moderate longitudinal trajectories.

**Conclusions:**

In this cohort of students in sport and health science programs, several key health indicators remained stable or showed favorable changes during undergraduate studies. Although VO₂peak increased in the unadjusted within-subject comparison, this increase was not attributable to an independent effect of time after adjustment for relevant covariates. Higher weekly training volume was independently associated with higher cardiorespiratory fitness but did not significantly moderate changes in VO₂peak over time.

**Supplementary Information:**

The online version contains supplementary material available at https://doi.org/10.1186/s12889-026-29582-5.

## Introduction

University years represent a critical developmental period characterized by new academic demands, increased autonomy, and substantial lifestyle changes. This phase is not only formative for cognitive and social development but also for the establishment of long-term health behaviors, highlighting universities as important settings for health promotion and disease prevention [1]. Previous studies consistently indicate that university students often exhibit suboptimal health profiles, including insufficient physical activity, unhealthy dietary patterns, elevated stress levels, and engagement in health-risk behaviors such as smoking and excessive alcohol consumption [2–4]. However, the predominance of cross-sectional designs limits conclusions regarding health trajectories during university education. Consequently, it remains unclear whether students’ physical health and fitness deteriorate, stabilize, or improve over time [5]. Longitudinal evidence is scarce, and findings are inconsistent, frequently relying on self-reported data or short observation periods [1]. Moreover, little is known about the role of modifiable factors, such as physical activity volume or the moderating effects of sex and age on health trajectories during early adulthood [1, 6].

Student health management provides a unique opportunity to address these gaps through repeated, objective health assessments within a university-based health promotion framework. A comprehensive physiological and clinical test battery enables longitudinal analyses of cardiorespiratory fitness, anthropometric measures, cardiovascular risk factors, and a structured medical history interview supplemented with questions on smoking status, alcohol consumption, weekly training volume, and dietary patterns in students enrolled in sport and health science programs.

Examining temporal changes in these markers is essential to determine whether the university period represents a phase of health decline or, conversely, a window for positive development. In addition, identifying behavioral determinants such as weekly training volume may help clarify which modifiable factors are associated with favorable trajectories and may therefore serve as targets for campus-based prevention strategies. Finally, investigating potential differences according to sex and age is necessary to establish whether health promotion approaches should be tailored to specific subgroups or can be implemented broadly across the student population.

Accordingly, the aim of this study is to examine longitudinal changes in key health indicators across the university study period. Specifically, the following research questions are addressed:

1. How do objectively measured cardiovascular health indicators and cardiorespiratory fitness develop during undergraduate studies in sport- and health-oriented students?
2. Which behavioral and demographic factors are associated with these longitudinal health trajectories?
3. Are longitudinal changes primarily explained by modifiable lifestyle factors (e.g., weekly training volume) rather than by demographic characteristics such as sex, age, or study program?

## Methods

### Study design and participants

**Fig. 1:**
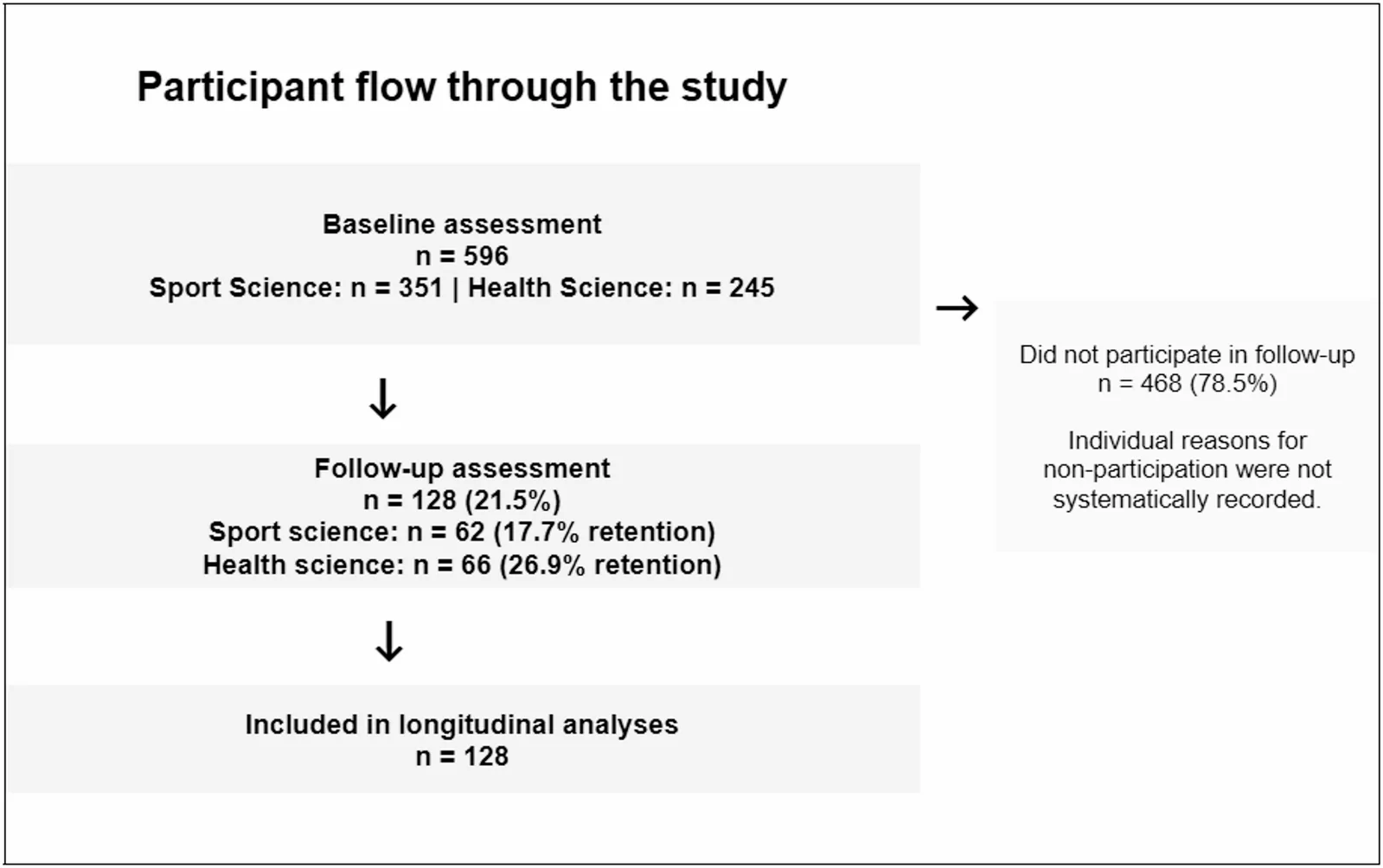
Participant flow from baseline assessment to follow-up and inclusion in the longitudinal analyses. Retention rates represent the proportion of baseline participants within each study program who completed the follow-up assessment. Individual reasons for non-participation at follow-up were not systematically recorded

This longitudinal observational study was conducted as part of a university-based health promotion initiative at the Technical University of Munich (TUM). Participants were undergraduate students enrolled in bachelor’s programs in sport science or health science. Of 596 students who completed the baseline assessment, 128 students (75% female) participated in the follow-up examination and were included in the longitudinal analyses (Fig. 1). Baseline assessments were conducted between May 2017 and July 2024 during the first or second semester of study, while follow-up assessments took place between October 2020 and June 2026 (semesters five to eight). The mean follow-up interval was 2.53 ± 0.60 years. Mean age was 21.0 ± 2.1 years at baseline and 23.5 ± 2.2 years at follow-up.

Participation was voluntary, limited to students aged ≥ 18 years, and required written informed consent. The study was approved by the Ethics Committee of TUM (Reference No. 379/19 S-SR) and conducted in accordance with the Declaration of Helsinki.

### Examination procedures

The assessment protocol has been described in detail in a previous publication [3]. Briefly, anthropometrics and body composition were assessed using multi-frequency bioelectrical impedance analysis (InBody 770; InBody Europe B.V., Germany). The variables analyzed comprised body height [cm], body weight [kg], body mass index (BMI) [kg/m²], body fat percentage [%], and muscle mass [kg]. Central blood pressure was measured with an oscillometric device (Mobil-O-Graph; I.E.M. GmbH, Germany). Resting heart rate and central systolic and diastolic blood pressure were included. Resting and exercise 12-lead ECG recordings were obtained using a standardized diagnostic system (Custo ergo diagnostic 300; Customed GmbH, Germany). Cardiopulmonary exercise testing (CPET) was performed on a cycle ergometer using a ramp protocol with an initial workload of 50 W and a continuous increment of 20 W per minute (Excalibur Sport; Lode B.V., The Netherlands) with breath-by-breath gas-exchange analysis (MetaLyzer 3B; Cortex Biophysik GmbH, Germany). VO₂peak was defined as the highest 30-second averaged value achieved during exercise. Transthoracic echocardiography was conducted by an experienced cardiologist using a GE Vivid E9 XDclear system (GE Healthcare GmbH, Germany).

A structured medical interview *(Supplement 1)* covered medical history and was supplemented with questions on smoking status, alcohol consumption, weekly training volume, and dietary patterns. Dietary patterns were recorded as omnivorous, vegetarian, or vegan. For the longitudinal analysis, vegetarian and vegan dietary patterns were combined into a single vegetarian/vegan category. Smoking status was recorded as a binary variable (yes/no). Participants reporting either regular or occasional smoking were classified as current smokers. For the longitudinal analysis, alcohol consumption was recorded as a binary variable indicating high-risk alcohol consumption (yes/no). High-risk alcohol consumption was defined as > 40 g/day in men or > 20 g/day in women on more than three days per week. Participants not meeting this criterion were classified as not having high-risk alcohol consumption (according to thresholds reported in the review of international alcohol-consumption guidelines [7], which was also used in the cross-sectional analysis previously published [3]). Participants were asked to report the habitual amount of structured exercise or training performed during a typical week, expressed in hours per week. The same standardized assessment procedure was applied at baseline and follow-up. This study-specific measure was intended to capture habitual structured training volume rather than total physical activity. It has not been independently validated against objective physical activity measures or formally compared with the IPAQ.

### Statistical analysis

All statistical analyses were conducted using R (Version 4.4.2, R Foundation for Statistical Computing, Vienna, Austria). Descriptive statistics are presented as mean ± standard deviation. Inferential statistical analyses were performed in two steps. First, longitudinal changes between baseline and follow-up were examined using paired-samples t-tests. Changes in paired binary variables between baseline and follow-up were analyzed using McNemar’s test. To assess the potential impact of selective attrition, an inverse probability of retention weighting (IPRW) sensitivity analysis was performed. The probability of participation in the follow-up assessment was estimated using binary logistic regression based on baseline characteristics associated with retention, including sex, study program, and baseline VO₂peak. Stabilized inverse probability weights were calculated from the predicted probabilities of follow-up participation and applied to the paired analyses of the main longitudinal outcomes. Weighted results were compared with the primary unweighted analyses across the total cohort to assess whether differential retention materially affected the observed longitudinal changes. Given the exploratory nature of the longitudinal assessment across multiple health domains, no formal adjustment for multiple testing was applied. Accordingly, p-values from individual and subgroup comparisons were interpreted descriptively and with caution.

Second, linear mixed-effects models (LMMs) were fitted to evaluate longitudinal changes while accounting for repeated measurements within individuals and adjusting for sex, age, study program, weekly training volume, and their interactions with time. Separate models were estimated for VO₂peak, central systolic blood pressure, muscle mass, body fat percentage, body weight, and BMI. Fixed effects included time (baseline vs. follow-up), sex, age, study program, weekly training volume [h/week], and interaction terms between time and each covariate. Age and weekly training volume were entered as continuous variables and were neither centered nor otherwise transformed. Sex and study program were entered as categorical variables. Participant-specific random intercepts were included to account for within-subject correlation. Random slopes for time were not included because this model specification resulted in an unidentifiable random-effects structure. Because time-by-covariate interactions were included, the main effect of time represents the estimated change from baseline to follow-up for the reference categories of the categorical predictors and at zero values of the continuous predictors. Models were estimated using restricted maximum likelihood (REML), and Satterthwaite-adjusted degrees of freedom were used for hypothesis testing. Model assumptions were checked visually and were considered adequately met for all outcomes. Statistical significance was defined as *p* < .05. Changes in categorical variables with two paired categories between baseline and follow-up were analyzed using McNemar’s test for paired nominal data.

## Results

### Participant characteristics

A total of 128 students (75% female) were included in the longitudinal analyses. Retention differed by study program. Of the 351 students enrolled in sport science at baseline, 62 (17.7%) completed the follow-up assessment, whereas 66 of 245 students in health science (26.9%) were retained. Accordingly, health science students were more likely to participate in follow-up, indicating selective attrition by study program. Baseline comparisons also revealed sex-differential retention, with women overrepresented in the longitudinal sample compared with the original cohort (75% vs. 63%). In contrast, men were more likely to be lost to follow-up. Overall, these findings suggest selective retention according to both study program and sex. Male participants (*n* = 32) had a mean height of 178.79 ± 7.86 cm and a mean body weight of 75.01 ± 10.97 kg at baseline and 76.30 ± 11.93 kg at follow-up. Female participants (*n* = 96) had a mean height of 166.48 ± 6.49 cm and a stable body weight (59.39 ± 7.48 kg at baseline; 59.49 ± 8.06 kg at follow-up). Baseline characteristics and follow-up values for the total cohort and stratified by sex are presented in Table 1.

**Table 1:** Baseline and follow-up characteristics of the total cohort and stratified by sex

Characteristics	Total Cohort			Male Participants			Female Participants		
	baseline	follow-up	p-value	baseline	follow-up	p-value	baseline	follow-up	p-value
* *p* < .05	*n* = 128	*n* = 128		*n* = 32	*n* = 32		*n* = 96	*n* = 96	
Age, y	20.97 ± 2.10	23.45 ± 2.25	< 0.001*	21.41 ± 2.53	24.03 ± 2.71	< 0.001*	20.82 ± 1.94	23.26 ± 2.05	< 0.001*
Height, cm	169.55 ± 8.67			178.79 ± 7.86			166.48 ± 6.49		
Weight, kg	63.30 ± 10.83	63.63 ± 11.56	0.340	75.01 ± 10.97	76.30 ± 11.93	0.073	59.39 ± 7.48	59.49 ± 8.06	0.967
BMI, kg/m²	21.88 ± 2.79	22.00 ± 2.58	0.322	23.32 ± 2.50	23.81 ± 2.91	0.036*	21.40 ± 2.00	21.40 ± 2.16	0.980
Body fat percentage, %	19.11 ± 6.57	19.51 ± 6.19	0.182	12.63 ± 6.36	14.37 ± 7.21	< 0.001*	21.27 ± 5.06	21.22 ± 4.73	0.871
Muscle mass, kg	28.60 ± 6.85	29.10 ± 6.93	0.006*	37.53 ± 7.12	37.38 ± 7.94	0.740	25.62 ± 3.21	26.34 ± 3.57	< 0.001*
Central systolic blood pressure, mmHg	110.03 ± 10.31	111.15 ± 11.35	0.158	113.97 ± 13.34	117.50 ± 13.71	0.090	108.72 ± 8.76	109.03 ± 9.64	0.696
Central diastolic blood pressure, mmHg	73.09 ± 7.95	73.19 ± 7.72	0.878	72.91 ± 8.69	75.09 ± 9.92	0.123	73.16 ± 7.74	72.55 ± 6.78	0.360
Resting heart rate, bpm	68.05 ± 11.53	64.80 ± 10.40	< 0.001*	68.16 ± 11.77	64.25 ± 10.04	0.022*	68.02 ± 11.51	64.98 ± 10.56	< 0.001*
Weekly training volume, h/week	6.11 ± 3.26	6.48 ± 3.48	0.105	7.51 ± 3.70	7.35 ± 4.36	0.734	5.64 ± 2.98	6.19 ± 3.11	0.042*
VO₂peak, ml·kg⁻¹·min⁻¹	43.59 ± 6.99	45.36 ± 7.68	< 0.001*	49.03 ± 7.12	49.66 ± 8.81	0.384	41.77 ± 5.96	43.93 ± 6.73	< 0.001*
Pmax, W	241.09 ± 54.03	244.58 ± 57.50	0.129	306.19 ± 52.75	304.34 ± 62.77	0.677	219.39 ± 33.04	224.66 ± 39.12	0.050

Baseline comparisons between participants retained for follow-up and those lost to follow-up revealed no significant differences in age, body weight, BMI, body fat percentage, muscle mass, or weekly training volume (all *p* > .05). No significant overall difference in baseline VO₂peak was observed between retained and non-retained participants. In sex-stratified analyses, however, retained male participants demonstrated higher baseline VO₂peak values, whereas retained female participants showed lower baseline VO₂peak values compared with those lost to follow-up (male mean difference = 4.68 ml·kg⁻¹·min⁻¹, *p* = .002, Cohen’s d = 0.432 and female mean difference = -2.44 ml·kg⁻¹·min⁻¹, *p* = .007, Cohen’s d = 0.342).

### Changes in cardiorespiratory fitness and hemodynamic measures

In the total cohort, VO₂peak increased significantly from 43.59 ± 6.99 to 45.36 ± 7.68 ml·kg⁻¹·min⁻¹ (*p* < .001, Cohen’s d = 0.434). Resting heart rate decreased from 68.05 ± 11.53 to 64.80 ± 10.40 bpm (*p* < .001, Cohen’s d = 0.378). Neither central systolic nor central diastolic blood pressure changed significantly. Body weight, BMI, and body fat percentage did not change significantly, whereas muscle mass increased from 28.60 ± 6.85 kg at baseline to 29.10 ± 6.93 kg at follow-up (*p* = .006, Cohen’s d = 0.246). No significant changes were observed for maximal power output (Pmax). Detailed results are shown in Table 1. The stabilized inverse probability of retention weights showed a mean of 0.98 (SD 0.29) and ranged from 0.67 to 1.74, with no indication of extreme weights. IPRW-weighted analyses yielded results consistent with the unweighted analyses. VO₂peak remained significantly increased (weighted mean change + 1.55 ml·kg⁻¹·min⁻¹, *p* < .001), muscle mass remained significantly increased (+ 0.43 kg, *p* = .032), and resting heart rate remained significantly decreased (− 3.42 bpm, *p* < .001). Thus, weighting for differential retention based on observed baseline characteristics did not materially alter the direction or statistical interpretation of the primary paired analyses.

### Sex-specific analyses

Male participants showed significant increases in body fat percentage (12.63 ± 6.36% to 14.37 ± 7.21%, *p* < .001, Cohen’s d = 0.642) and body mass index (23.32 ± 2.50 kg/m² to 23.81 ± 2.91 kg/m², *p* = .036, Cohen’s d = 0.387). Muscle mass decreased and body weight increased, but neither change was statistically significant. Weekly training volume also decreased without reaching statistical significance, whereas resting heart rate decreased significantly (68.16 ± 11.77 bpm to 64.25 ± 10.04 bpm, *p* = .022, Cohen’s d = 0.425). VO₂peak and Pmax showed numerical increases, but neither reached statistical significance. Neither central systolic nor central diastolic blood pressure changed significantly (Table 1).

Female participants demonstrated a significant increase in muscle mass (25.62 ± 3.21 kg to 26.34 ± 3.57 kg, *p* < .001, Cohen’s d = 0.391), while body fat percentage, BMI, and body weight remained stable. VO₂peak increased significantly (41.77 ± 5.96 ml·kg⁻¹·min⁻¹ to 43.93 ± 6.73 ml·kg⁻¹·min⁻¹, *p* < .001, Cohen’s d = 0.531), accompanied by a significant reduction in resting heart rate (68.02 ± 11.51 bpm to 64.98 ± 10.56 bpm, *p* < .001, Cohen’s d = 0.360) and a significant increase in weekly training volume (5.64 ± 2.98 h to 6.19 ± 3.11 h, *p* = .042, Cohen’s d = 0.211). Neither central systolic nor central diastolic blood pressure changed significantly (Table 1).

### Study program–specific analyses

Students enrolled in the Bachelor of Science in sport science showed no significant changes in body weight or body composition. This group demonstrated a significant increase in VO₂peak (45.68 ± 7.28 to 47.79 ± 8.04 ml·kg⁻¹·min⁻¹, *p* < .001, Cohen’s d = 0.524) and a significant reduction in resting heart rate (67.02 ± 10.60 to 64.02 ± 9.34 bpm, *p* = .005, Cohen’s d = 0.372). Weekly training volume increased slightly, but the change was not statistically significant, and Pmax remained unchanged. Neither central systolic nor central diastolic blood pressure changed significantly (Table 2).

**Table 2:** Baseline and follow-up characteristics of the total cohort stratified by study program

Characteristics	Total Cohort			B.Sc. Sport science			B.Sc. Health science		
	baseline	follow-up	p-value	baseline	follow-up	p-value	baseline	follow-up	*p-value*
* *p* < .05	*n* = 128 (25% male)	*n* = 128 (25% male)		*n* = 62 (32% male)	*n* = 62 (32% male)		*n* = 66 (18% male)	*n* = 66 (18% male)	
Age, y	20.97 ± 2.10	23.45 ± 2.25	< 0.001*	20.61 ± 1.78	23.06 ± 1.99	< 0.001*	21.30 ± 2.35	23.82 ± 2.42	< 0.001*
Height, cm	169.55 ± 8.67			170.90 ± 8.17			168.29 ± 8.99		
Weight, kg	63.30 ± 10.83	63.63 ± 11.56	0.340	64.31 ± 10.33	64.31 ± 10.77	0.991	62.34 ± 11.27	62.99 ± 12.30	0.223
BMI, kg/m²	21.88 ± 2.79	22.00 ± 2.58	0.322	21.87 ± 2.15	21.87 ± 2.27	0.956	21.87 ± 2.42	22.13 ± 2.86	0.180
Body fat percentage, %	19.11 ± 6.57	19.51 ± 6.19	0.182	17.60 ± 6.45	17.80 ± 5.73	0.629	20.53 ± 6.41	21.11 ± 6.22	0.175
Muscle mass, kg	28.60 ± 6.85	29.10 ± 6.93	0.006*	29.69 ± 6.57	30.04 ± 6.54	0.080	27.58 ± 6.99	28.22 ± 7.21	0.035*
Central systolic blood pressure, mmHg	110.03 ± 10.31	111.15 ± 11.35	0.158	110.06 ± 10.06	111.39 ± 10.92	0.209	110.00 ± 10.62	111.92 ± 11.83	0.437
Central diastolic blood pressure, mmHg	73.09 ± 7.95	73.19 ± 7.72	0.878	72.52 ± 7.81	73.29 ± 7.95	0.364	73.64 ± 8.11	73.09 ± 7.56	0.532
Resting heart rate, bpm	68.05 ± 11.53	64.80 ± 10.40	< 0.001*	67.02 ± 10.60	64.02 ± 9.34	0.005*	69.03 ± 12.34	65.53 ± 11.32	0.003*
Weekly training volume, h/week	6.11 ± 3.26	6.48 ± 3.48	0.105	7.40 ± 3.36	7.69 ± 3.58	0.402	4.89 ± 2.66	5.35 ± 3.00	0.149
VO₂peak, ml·kg⁻¹·min⁻¹	43.59 ± 6.99	45.36 ± 7.68	< 0.001*	45.68 ± 7.28	47.79 ± 8.04	< 0.001*	41.62 ± 6.14	43.08 ± 6.61	0.006*
Pmax, W	241.09 ± 54.03	244.58 ± 57.50	0.129	255.31 ± 53.53	259.42 ± 57.59	0.161	227.73 ± 51.39	230.64 ± 54.23	0.411

Students in the Bachelor of Science in health science exhibited a significant increase in muscle mass (27.58 ± 6.99 to 28.22 ± 7.21 kg, *p* = .035, Cohen’s d = 0.266). In contrast, body weight, body fat percentage, and BMI did not change significantly. VO₂peak increased significantly (41.62 ± 6.14 to 43.08 ± 6.61 ml·kg⁻¹·min⁻¹, *p* = .006, Cohen’s d = 0.351), and resting heart rate decreased significantly (69.03 ± 12.34 to 65.53 ± 11.32 bpm, *p* = .003, Cohen’s d = 0.383). Weekly training volume and Pmax increased numerically, but neither change was statistically significant. Neither central systolic nor central diastolic blood pressure changed significantly (Table 2).

### Lifestyle changes and additional individual risk assessments

The prevalence of current smoking decreased significantly from 10.2% (*n* = 13/128) at baseline to 3.9% (*n* = 5/128) at follow-up (McNemar’s *p* = .025). The proportion of participants classified as having high-risk alcohol consumption decreased from 13.3% (*n* = 17/128) at baseline to 6.3% (*n* = 8/128) at follow-up (McNemar’s *p* = .046). In contrast, adherence to a vegetarian or vegan diet increased from 35.2% (*n* = 45/128) to 43.0% (*n* = 55/128) over the study period (McNemar’s *p* = .007) (Table 3).

**Table 3:** Changes in smoking status, alcohol consumption, and dietary patterns between baseline and follow-up

Lifestyle characteristic	Baseline (t0)		Follow-up (t1)		*p*-value
*n* = 128		*n*		*n*	
Current smoking	10.2%	13	3.9%	5	0.025*
Alcohol consumption ♂ > 40 g/day, ♀ > 20 g/day, > 3 d/week	13.3%	17	6.3%	8	0.046*
Vegetarian/vegan diet	35.2%	45	43.0%	55	0.007*

* *p* < .05

### Longitudinal trajectories

Linear mixed-effects models were used to examine longitudinal changes in cardiorespiratory fitness, central systolic blood pressure, and anthropometric outcomes, while accounting for repeated measurements within individuals and adjusting for sex, age, study program, and weekly training volume. Time-by-covariate interaction terms were included to assess whether longitudinal trajectories differed according to sex, age, study program, or weekly training volume.

After adjustment for the included covariates, no significant main effect of time was observed for VO₂peak (b = 6.00, SE = 3.88, *p* = .124), central systolic blood pressure (b = 6.14, SE = 9.12, *p* = .502), body weight (b = -4.90, SE = 4.00, *p* = .223), BMI (b = -1.59, SE = 1.43, *p* = .270), body fat percentage (b = 0.25, SE = 3.27, *p* = .940), or muscle mass (b = -2.93, SE = 2.06, *p* = .159).

Weekly training volume was a significant positive predictor of VO₂peak (b = 0.51, SE = 0.13, *p* < .001) and was inversely associated with body fat percentage (b = -0.30, SE = 0.11, *p* = .007). However, weekly training volume did not significantly moderate longitudinal changes in any outcome (all time × training volume interactions *p* > .05).

Female sex was associated with lower baseline VO₂peak (b = -6.01, SE = 1.33, *p* < .001), lower central systolic blood pressure (b = -5.65, SE = 2.25, *p* = .013), lower body weight (b = -15.20, SE = 1.84, *p* < .001), lower BMI (b = -1.87, SE = 0.48, *p* < .001), and lower muscle mass (b = -11.50, SE = 0.99, *p* < .001), while body fat percentage was higher in women than in men (b = 8.19, SE = 1.09, *p* < .001). No significant moderation of longitudinal change by sex was observed for any outcome except body fat percentage, for which the time × sex interaction reached statistical significance (b = -1.55, SE = 0.69, *p* = .026).

Age was positively associated with body fat percentage (b = 0.41, SE = 0.21, *p* = .048), whereas no significant associations with the remaining baseline outcomes were observed. Furthermore, none of the time × age interactions reached statistical significance (all *p* > .05). Study program neither predicted baseline levels nor moderated longitudinal changes across any outcome variable (all *p* > .05).

## Discussion

This longitudinal study examined changes in cardiorespiratory fitness, anthropometric parameters, blood pressure, and cardiovascular risk indicators among students enrolled in sport and health science programs throughout their undergraduate studies. In the first analytical step, paired t-tests were used to evaluate unadjusted within-subject changes between baseline and follow-up. While this approach provides a straightforward comparison of mean differences, it does not account for potential confounding variables or inter-individual variability. Therefore, in a second step, linear mixed-effects models were applied to investigate longitudinal trajectories while adjusting for relevant covariates and accounting for correlations among repeated measurements. Compared with paired t-tests, mixed-effects models provide a more comprehensive assessment of longitudinal changes by accounting for covariates and within-subject correlation.

The principal findings were threefold. First, unadjusted analyses indicated improvements in cardiorespiratory fitness, reflected by increased VO₂peak and reduced resting heart rate. However, after adjustment for sex, age, study program, and weekly training volume in the mixed-effects models, no independent longitudinal effect of time on VO₂peak remained. Second, although muscle mass showed a small but statistically significant increase in the unadjusted paired analysis, no independent main effect of time was observed for muscle mass or the other anthropometric outcomes in the adjusted mixed-effects models. Third, weekly training volume was positively associated with baseline VO₂peak, whereas there was no evidence that weekly training volume, sex, age, or study program moderated longitudinal trajectories. Overall, these findings indicate that cardiorespiratory fitness was associated with individual characteristics and habitual training behavior, while no statistically significant independent main effect of time was observed after adjustment.

Although paired analyses demonstrated a significant increase in VO₂peak, no statistically significant independent main effect of time was observed after adjustment for relevant covariates in the mixed-effects model. This indicates that the unadjusted within-subject increase should not be interpreted as an independent effect of time during university studies. Weekly training volume was positively associated with baseline VO₂peak, indicating that participants reporting higher habitual training volumes tended to exhibit higher cardiorespiratory fitness. However, the time × training volume interaction was not statistically significant, providing no evidence that weekly training volume moderated the longitudinal change in VO₂peak. Given the observational design, these associations should not be interpreted as evidence of mediation or causality. The observed reduction in resting heart rate nevertheless supports the notion of maintained cardiovascular fitness within the cohort. This finding contrasts with previous reports of unfavorable changes in selected health behaviors and health indicators during the transition to and progression through university education [1, 8]. Importantly, these changes are unlikely to be explained by chronological aging alone, as age neither predicted baseline VO₂peak nor moderated longitudinal trajectories. Age-related declines in cardiorespiratory fitness are generally less pronounced in younger adults and become progressively more evident with increasing age [9]. Given the observational design, causality cannot be inferred, and the possibility of measurement familiarity, behavioral self-selection, or selective retention contributing to the observed changes cannot be excluded.

Instead, weekly training volume emerged as an important behavioral correlate of baseline VO₂peak in the present cohort. This finding is consistent with the established role of regular physical activity and exercise training in supporting higher levels of cardiorespiratory fitness [10]. However, weekly training volume did not significantly moderate longitudinal changes in VO₂peak, indicating that participants with higher reported training volumes did not demonstrate significantly different trajectories over the observation period. From a preventive perspective, the findings nevertheless support the importance of maintaining regular physical activity during university studies, while the observational design precludes causal inference.

Beyond physical activity, several lifestyle behaviors also changed favorably during the observation period. Smoking prevalence decreased by more than half, high-risk alcohol consumption became less frequent, and adherence to vegetarian or vegan dietary patterns increased. Although these variables were not included as predictors in the mixed-effects models, and causal inferences cannot be drawn, these findings suggest that health-related behaviors may have improved more broadly during the university years. Together with stable anthropometric measures and maintained cardiorespiratory fitness, these observations support the notion that students in sport- and health-related degree programs may adopt or maintain healthier lifestyles over time. These favorable behavioral changes contrast with reports indicating that health-risk behaviors remain common in broader university populations [4, 11].

Students who choose sport- and health-related degree programs may already enter university with above-average interest in physical activity and health-promoting behaviors. The baseline VO₂peak values were at the lower end of reported reference values for sport and exercise science students. For example, Paradisis et al. reported a mean VO₂peak of 49.98 ± 8.33 ml·kg⁻¹·min⁻¹ in a physical education cohort, with values of 52.35 ± 6.25 ml·kg⁻¹·min⁻¹ in men and 47.42 ± 5.62 ml·kg⁻¹·min⁻¹ in women [12]. In contrast, lower aerobic capacity has been reported in medical student cohorts, typically around 38–45 ml·kg⁻¹·min⁻¹ in men and 30–38 ml·kg⁻¹·min⁻¹ in women [13]. These differences likely reflect varying levels of habitual physical activity, training exposure, and self-selection into physically active study programs.

In addition, the university setting itself may have supported continued engagement in exercise through accessible sports facilities, a physically active peer environment, and integration of health-related topics into academic life. These explanations remain speculative, as environmental and motivational determinants were not directly assessed, but they provide plausible hypotheses for future research.

Sex significantly predicted baseline VO₂peak, with women demonstrating lower values than men, consistent with established physiological differences in cardiorespiratory fitness. However, sex did not moderate longitudinal trajectories, indicating that both sexes followed comparable patterns of change over time after adjustment for relevant covariates. This discrepancy between the sex-stratified paired analyses and the adjusted mixed-effects models illustrates the importance of accounting for baseline differences and individual variability when evaluating longitudinal data.

At the cohort level, body weight, BMI, and body fat percentage remained largely stable across the observation period, whereas muscle mass showed a small but statistically significant increase in the unadjusted paired analysis. However, this increase was not supported by a significant independent main effect of time in the adjusted mixed-effects model. This contrasts with reports of gradual weight gain and unfavorable body composition changes during university years in more heterogeneous student populations [6, 14]. The absence of significant longitudinal effects in the mixed-effects models indicates that adverse anthropometric changes were not evident during university attendance in this cohort. Rather, body composition trajectories in early adulthood are likely shaped by lifestyle behaviors such as physical activity, dietary habits, and overall energy balance. Whether the observed stability in the present sample reflects self-selection into health-oriented study programs, supportive environmental conditions, or both cannot be determined conclusively.

The stability of anthropometric measures suggests that adverse changes in body composition were largely absent in the present cohort. However, cardiovascular health cannot be fully captured by anthropometric indicators alone. To provide a more comprehensive assessment of health trajectories during university years, changes in central systolic blood pressure were also examined. Central systolic blood pressure remained largely stable over the observation period, with no statistically significant change observed in the paired analysis and no significant longitudinal effect detected in the adjusted mixed-effects model. These consistent findings suggest that central systolic blood pressure did not change substantially during the observed period in this cohort. Longitudinal epidemiological studies demonstrate heterogeneous blood pressure trajectories from young adulthood onward, including both stable and progressively increasing patterns [15]. Against this background, the stability observed in the present cohort was accompanied by stable anthropometric measures and relatively high reported levels of habitual training.

From a public health perspective, the findings underline the potential of universities as settings for preventive health interventions. The combination of repeated objective assessments, longitudinal monitoring, and easy access to opportunities for physical activity may support favorable health trajectories during a life phase often considered vulnerable to behavioral decline. Although the adjusted analyses did not demonstrate an independent effect of time on cardiorespiratory fitness, the positive association between habitual training volume and VO₂peak emphasizes the importance of maintaining regular physical activity throughout university studies. The observation period also encompassed the COVID-19 pandemic, and changes in access to sports facilities, organized exercise, university attendance, and daily routines may have affected individual physical activity patterns. Accordingly, the observed trajectories should be interpreted within this heterogeneous temporal context, and potential pandemic-related, calendar, and cohort effects cannot be excluded. The observed reductions in smoking and high-risk alcohol consumption, together with the increasing adoption of vegetarian or vegan diets, further indicate that favorable behavioral changes may accompany university-based health promotion initiatives in health-oriented student populations. Extending similar approaches to student groups beyond sport- and health-related disciplines may therefore represent a promising strategy for broader health promotion.

### Limitations

Several limitations should be considered. First, selection bias is likely, as participation was voluntary and restricted to students from two health-oriented study programs. Accordingly, the cohort cannot be considered representative of the general student population. Second, retention at follow-up was selective, with higher participation among women and health science students, whereas male students and sport science students were more frequently lost to follow-up. Baseline comparisons between retained and non-retained participants showed no significant differences in age, body weight, BMI, body fat percentage, muscle mass, or weekly training volume. Although no significant overall difference in baseline VO₂peak was observed, sex-stratified analyses indicated differential retention patterns, with retained male participants demonstrating higher baseline VO₂peak values compared with non-retained participants. Selective attrition may therefore have influenced the observed longitudinal trajectories and may limit the generalizability of the findings. To address the potential impact of selective attrition, an IPRW sensitivity analysis was performed for the total cohort. The weighted estimates were comparable to the unweighted analyses and did not alter the direction or statistical interpretation of the main findings, providing some reassurance that differential retention related to the measured baseline characteristics did not substantially drive the observed longitudinal changes. However, IPRW can only account for selection associated with observed variables included in the retention model, and residual selection bias due to unmeasured determinants of follow-up participation cannot be excluded. Accordingly, the longitudinal findings should primarily be interpreted as applying to the retained cohort and should not be considered representative of the broader university student population. Third, the modest sample size may have limited statistical power to detect smaller subgroup-specific moderation effects. Fourth, the extended recruitment and follow-up period overlapped with the COVID-19 pandemic, thereby introducing potential temporal heterogeneity. Baseline and follow-up assessments were conducted over multiple calendar years, meaning that participants were exposed to different academic, social, and public health conditions during their respective observation periods. Pandemic-related restrictions temporarily affected university life, organized sports, access to sports facilities, and opportunities for habitual physical activity, all of which may have influenced training behavior and physiological outcomes. The impact of the pandemic could not be adequately represented by a simple binary pandemic-period indicator because the nature and extent of restrictions varied substantially over time, and individual exposure depended on the timing of baseline and follow-up assessments. Furthermore, pandemic-related effects cannot be clearly separated from broader calendar or cohort effects in the present observational design. These temporal factors were therefore not explicitly included in the statistical models. Consequently, unmeasured pandemic-related, calendar, or cohort effects may have contributed to the observed longitudinal patterns, and their direction and magnitude cannot be determined from the present data. Fifth, weekly training volume was assessed by self-report during a structured interview rather than by objective activity monitoring. The measure may therefore be subject to recall and social desirability bias and may not fully capture the actual duration, intensity, or temporal variability of training. Furthermore, this study-specific measure has not been independently validated against objective physical activity measures or formally compared with established questionnaires such as the IPAQ. The observed association between weekly training volume and VO₂peak should therefore be interpreted with appropriate caution.

Despite these limitations, the study provides rare longitudinal evidence based on objective physiological assessments in university students.

Given the predominance of cross-sectional data in this field, these findings contribute important insights into the heterogeneity of student health trajectories and identify weekly training volume as a relevant correlate of cardiorespiratory fitness.

## Conclusion

Students enrolled in sport and health science programs entered university with comparatively favorable baseline health profiles and generally maintained key health indicators throughout their undergraduate studies. In unadjusted paired analyses, VO₂peak and muscle mass increased and resting heart rate decreased, while anthropometric measures remained largely stable. However, the mixed-effects model showed no statistically significant independent main effect of time on VO₂peak after adjustment for sex, age, study program, and weekly training volume. Higher weekly training volume was independently associated with higher cardiorespiratory fitness, whereas sex, age, and study program did not significantly moderate longitudinal VO₂peak trajectories.

These findings suggest that unfavorable health trajectories during university years may not be inevitable in health-oriented student populations. Rather than indicating a uniform improvement in cardiorespiratory fitness over time, the results highlight the importance of habitual training behavior and individual characteristics for fitness levels during undergraduate studies. Future research should examine which environmental, behavioral, and institutional factors most effectively support favorable health trajectories across diverse student populations.

## Supplementary Information

Supplementary Material 1.

## Data Availability

The datasets generated and/or analyzed during the current study are not publicly available, as they contain sensitive participant data and are owned by the Technical University of Munich (TUM) as part of the TUM4Health project and the Student Health Management Program. The datasets are available from the corresponding author upon reasonable request, subject to approval by the Technical University of Munich and the completion of an appropriate data use and confidentiality agreement in accordance with applicable data protection regulations.
