# Supplementary material for: From freshman to graduate: longitudinal analysis of student health status in sport and health sciences
Source: BMC Public Health. 2026 Sep 21;26:2637. doi: 10.1186/s12889-026-29582-5 (PMC13591675; doi:10.1186/s12889-026-29582-5)
Supplement: Supplementary file 1 [file 12889_2026_29582_MOESM1_ESM.pdf]

Prevention and Performance Lab

Chair of Preventive Pediatrics
School of Medicine and Health
Technical University of Munich (TUM)

Gesund durchs Studium

Sports Medicine Examination Form

PID	Date	Date of Birth

Administrative

☐ Informed consent

Examinations Performed

☐ Anthropometry	☐ Resting ECG
☐ Blood pressure	☐ Pulse wave analysis
☐ Pulmonary function testing	☐ Medical history
☐ Physical examination	☐ Echocardiography
☐ Intima Media Thickness IMT	☐ Cardiopulmonary exercise testing (CPET)

Further Examinations Required

☐ 24-hour blood pressure monitoring ☐ 24-hour ECG ☐ Other: ______________________

Comments / Further Actions

Gesund durchs Studium

Anthropometry & Cardiovascular Assessment

Anthropometric Data

Parameter	Value	Unit
Height		cm
Weight (BIA)		kg
BMI (BIA)		kg/m^2
Body fat (BIA)		%
Muscle mass (BIA)		kg

Blood Pressure

Measurement	Systolic	Diastolic	Notes
1st (Mobil-O-Graph)<br>Left arm mm/Hg			
2nd (Mobil-O-Graph)<br>Left arm mm/Hg			
Central blood pressure (Mobil-O-Graph)<br>Left arm mm/Hg			

Pulse Wave Velocity (left arm): ________ m/s

Manual blood pressure (right arm): ______________ mmHg

Heart rate at rest: ________ bpm

Resting ECG

☐ No abnormal findings ☐ Abnormal findings: ____

Exercise ECG

☐ No abnormal findings ☐ Abnormal findings: ____

Pulmonary Function Testing

☐ No abnormal findings ☐ Abnormal findings: ____

Cardiopulmonary Exercise Testing

☐ No abnormal findings ☐ Abnormal findings: ____

Gesund durchs Studium

Medical History & Sports History

How are you feeling today?

___

Current complaints: ☐ None ☐ Yes: ______________________________

Family Situation

Sports History

Type of sport(s)	
Competitive athlete?	☐ No ☐ Yes
If yes, discipline	
If yes, since	
Training per week over all	________ hours
General physical activity/day	☐ Low ☐ Moderate ☐ High

Symptoms During Exercise

Shortness of breath	☐ No ☐ Yes
Dizziness / Syncope	☐ No ☐ Yes
Palpitations	☐ No ☐ Yes
Pain	☐ No ☐ Yes

Gesund durchs Studium
Personal Medical History

Past Medical History

Condition	No	Yes
Cardiovascular disease	☐	☐
Bronchial asthma	☐	☐
Diabetes mellitus	☐	☐
Thyroid disease	☐	☐
Seizure disorder / Epilepsy	☐	☐
Orthopedic disorders	☐	☐
Allergies	☐	☐
Childhood diseases	☐	☐
Injuries / Surgical procedures	☐	☐
Developmental delay	☐	☐
Other medical conditions	☐	☐

Vaccination status (according to STIKO)	☐ Complete ☐ Incomplete
Current medication ☐ None	

Gesund durchs Studium

Lifestyle, Nutrition & Pulmonary Function, Family History

Lifestyle

Dietary supplements ☐ None	
Alcohol consumption	☐ No<br>☐ Yes<br><br>____________________g/d<br><br>_____________________d/week
Smoking / Nicotine	☐ No ☐ Yes
Recreational drugs	☐ No ☐ Yes
Mood changes (depression, stress)	☐ No ☐ Yes
Nutrition	☐ Omnivorous<br>☐ Vegetarian<br>☐ Vegan<br>☐ else
Eating disorder	☐ No ☐ Yes

Preventive Examinations

Last dental examination	
Last ophthalmological examination	

Gynaecological History (if applicable)

Last gynaecological examination	
Menarche	
Menstrual cycle history	

Pulmonary Function Testing

Measurement	IVC	FVC	FEV1	FEV1%VC
Measurement 1				
Measurement 2				

Family History

Condition	No	Yes
Unexplained deaths	☐	☐
Sudden cardiac death	☐	☐
Disorders of lipid metabolism	☐	☐
Cardiovascular disease	☐	☐
Hypertension	☐	☐
Diabetes mellitus	☐	☐
Psychiatric disorders	☐	☐
Malignant disease	☐	☐
Marfan syndrome	☐	☐
Other medical conditions	☐	☐

Parental Height

Mother	_____________ cm
Father	_____________ cm

Physical Examination

Examination	Normal	Findings
Eyes	☐	
Ear, Nose and Throat (ENT)	☐	
Oral cavity / Teeth	☐	
Skin	☐	
Lungs	☐	
Heart / Vascular system	☐	
Abdomen	☐	
Musculature	☐	
Joints	☐	
Spine	☐	
Shoulders / Pelvis	☐	
Feet	☐	
Neurological examination	☐	
Genital examination	☐	

Gesund durchs Studium
Echocardiography Report

Cardiac Dimensions and Function

Parameter	Value	Comments / Z-score
Left atrium (LA)		
Right ventricular diameter (RVDd)		
IVS thickness (IVSd)		
LV internal diameter diastole (LVIDd)		
LV posterior wall thickness (LVPWd)		
LV internal diameter systole (LVIDs)		
Fractional shortening (FS)		
Ejection fraction (EF)		
Relative wall thickness		
Ascending aorta		
RV function / TAPSE		

Valve Assessment

Mitral valve: Mitral regurgitation ☐ Yes ☐ No Mitral valve prolapse ☐ Yes ☐ No

Tissue Doppler: E-wave ___ cm/s A-wave ___ cm/s E' lateral ___ E' medial ___ E/A ___ E/E' lateral ___ E/E' medial ___

Tricuspid valve: Tricuspid regurgitation ☐ Yes ☐ No Max gradient _____ mmHg

Aortic valve: Aortic regurgitation ☐ Yes ☐ No Tricuspid valve morphology ☐ Yes ☐ No

Pulmonary valve: Pulmonary regurgitation ☐ Physiological ☐ Pathological ☐ None

Intracardiac shunts ☐ Yes ☐ No Aortic arch ☐ Normal ☐ Abnormal

Coronary artery origins visualized: LCA _____ RCA _____

Overall Echocardiographic Findings

____
